# Pyroptosis-Related Signature and Tumor Microenvironment Infiltration Characterization in Head and Neck Squamous Cell Carcinoma

**DOI:** 10.3389/fcell.2022.702224

**Published:** 2022-05-31

**Authors:** Zeng-Hong Wu, Bian Wu, Cheng Li, You-Jing Zhang, Tao Zhou

**Affiliations:** ^1^ Department of Otorhinolaryngology, Union Hospital, Tongji Medical College, Huazhong University of Science and Technology, Wuhan, China; ^2^ Cancer Center, Union Hospital, Tongji Medical College, Huazhong University of Science and Technology, Wuhan, China; ^3^ Department of Otolaryngology Head and Neck Surgery, The Central Hospital of Wuhan, Tongji Medical College Huazhong University of Science and Technology, Wuhan, China; ^4^ School of Public Health, Tongji Medical College, Huazhong University of Science and Technology, Wuhan, China

**Keywords:** head and neck squamous cell carcinoma, pyroptosis, tumor microenvironment, TCGA, data mining

## Abstract

**Background:** Head and neck squamous cell carcinoma (HNSCC) is the sixth most widespread and deadly cancer. Until now, very few studies have systematically evaluated the role of pyroptosis-related genes (PRGs) and lncRNAs in HNSCC patients.

**Methods:** We integrated the genomic data to comprehensively assess the role of pyroptosis with the tumor microenvironment cell-infiltrating characteristics in HNSCC. In addition, we also constructed a set of the scoring system to calculate the pyroptosis dysfunction in each patient.

**Results:** The analysis of the CNV alteration frequency displayed that CNV changes were common in 33 PRGs, and the frequency of copy number gain and loss was similar. CASP8 demonstrated the highest mutation frequency. Considering the individual heterogeneity, a scoring system to quantify the pyroptosis pattern in each patient was constructed based on these phenotypic-related genes, which we named as the PyroptosisScore. The results indicated that the low PyroptosisScore group experienced increased extensive TMB than the high group, with the most significant mutated genes being TP53 and TTN. Finally, we tried to find some useful pyroptosis-related lncRNAs, and 14 differentially expressed lncRNAs were selected as independent prognosis factors of HNSCC patients based on the multivariate Cox analysis.

**Conclusion:** This work suggests the pyroptosis features and the potential mechanisms of the tumor microenvironment. The exploration may assist in identifying novel biomarkers and help patients predict prognosis, clinical diagnosis, and management.

## Background

Head and neck squamous cell carcinoma (HNSCC) arises in the stratified mucosa that lines the upper aero-digestive tract such as the tongue, lips, tonsil, nasopharynx, and larynx (Peyrade et al., 2021). There are over 655,000 new HNSCC cases per year, with over half a million deaths and ranking as the sixth most widespread and deadly cancer worldwide (Ferlay et al., 2010). The important danger elements involve tobacco smoking, alcohol, and high-risk human papillomavirus infection (Geng et al., 2021). Epstein–Barr virus infection is closely linked to nasopharyngeal carcinoma (Hitt et al., 1989). Although many forms of treatment, such as surgery, radiation, and chemotherapy, the asymptomatic nature and the absence of timely detection result in a 5-year survival rate of less than 50% in cancer patients (Bishop et al., 2021). Moreover, 50–60% of patients with over-stage III disease relapse locoregionally (Jacobs et al., 1992). Hence, an improved understanding of preclinical models is critical for determining prognosis between tumors and their host microenvironment and thus developing an appropriate therapeutic method.

Pyroptosis, also known as cell inflammatory necrosis, is a kind of programmed cell death, which is manifested by the continuous expansion of cells until the cell membrane ruptures, which causes the release of cell contents and activates a strong inflammatory response (Rühl et al., 2018). The body can recruit and activate caspase-1 protein through the classical inflammasome pathways (canonical inflammasome pathways) or activate the caspase 4/5/11 protein through the non-canonical inflammasome pathway to directly cleave and activate gasdermin protein D (GSDMD), eventually resulting in the formation of membrane pores and cell death (Jorgensen et al., 2017), while the gasdermin protein is the sole executor of cell pyroptosis. The GSDM protein family includes six members (GSDMD, GSDMA, GSDMB, GSDMC, GSDME (DFNA5), and DFNB59), which can be cleaved by cysteine protease caspase through a variety of ways, exposing its active N-terminus, and then, pores are formed on the cell membrane surface, causing cell swelling, thereby inducing cell pyroptosis and ultimately participating in the occurrence and development of inflammation and malignant tumors (Qiu et al., 2017). Lots of evidence shows pyroptosis in tumors is on the ascendancy. The study indicated that granzyme B can straightly cleave GSDME and initiate pyroptosis, promote activating the antitumor immune response, and suppress tumor growth (Zhou et al., 2020). Exogenous calcium treatment can result in a significant increase in the caspase-1 activity, and the N-terminal effector domain of GSDMD was exceedingly increased in the *CD38*-overexpressing HNSCC (Zhang et al., 2020). The study also found that the suppression of caspase-3-mediated GSDME-derived apoptosis contributed to noncancerous tissue defense in patients with oral squamous cell carcinoma among cisplatin chemotherapy (Huang et al., 2020). Interestingly, the study indicated knocking down LncRNA-XIST increased ROS levels and activated NLRP3 inflammasome in lung cells (Liu et al., 2019), and the generation of ROS was reported to induce pyroptosis (Cui et al., 2019). However, until now very few studies have systematically evaluated the role of pyroptosis-related genes (PRGs) and lncRNAs in HNSCC patients. In the current study, we integrated the genomic data to comprehensively assess the role of pyroptosis with the tumor microenvironment (TME) cell-infiltrating characteristics in HNSCC. In addition, we also constructed a set of the scoring system to calculate the pyroptosis dysfunction in each patient.

## Materials and Methods

### Data Collection

RNA-sequence (487 tumors and 42 normal, FPKM value) data based on 511 patients and the somatic mutation data were collected from The Cancer Genome Atlas (TCGA-HNSCC) database. Meanwhile, the GSE65858 cohorts were downloaded from Gene Expression Omnibus (GEO) in this study for further analysis. The clinical information of the patients is demonstrated in Table 1. The FPKM value is converted to transcripts per kilobase million (TPM) value. The batch effect from the non-biotech bias is corrected through the “combat” algorithm based on the SVA R package. The copy number variation (CNV) and long non-coding RNA (lncRNA) data were acquired from the TCGA database. We extracted 33 PRGs from prior reviews (Karki and Kanneganti, 2019; Xia et al., 2019; Wang and Yin, 2017; Man and Kanneganti, 2015), which are shown in Supplementary Table S1. GTF files were got from Ensembl for annotation to identify the lncRNAs and mRNAs. The connection was regarded as significant if the correlation coefficient |*R* (Ferlay et al., 2010)|>0.3 and *p* < 0.001 between mRNAs and lncRNAs. The differential expression of lncRNAs or mRNAs was decided at FDR<0.05 and |log_2_FC|≥1 using R software, limma package.

**TABLE 1:** Characteristics of HNSCC patients in TCGA and GEO.

Variable	Number of Samples	
Project	TCGA-HNSCC	GSE65858
Gender		
Male/Female	374/137	223/47
Age at diagnosis		
≤65/>65/NA	336/174/1	184/86
Grade		
G1/G2/G3/G4/NA	60/303/118/7/23	NA
Stage		
I/II/III/IV/NA	27/75/81/256/72	18/37/37/178
T		
T1/T2/T3/T4/NA	49/140/99/161/61	35/80/58/97
M		
M0/M1/NA	184/1/326	263/7/NA
N		
N0/N1/N2/N3/NA	172/67/167/8/97	94/31/132/13/NA

### Unsupervised Clustering for 33 PRGs

Based on the expressions of 33 PRGs, unsupervised cluster analysis was performed to identify different pyroptosis modification patterns, and patients were classified for further analysis. The number and stability of clustering are determined by using a consensus clustering algorithm (Hartigan, 1979). ConsensusClusterPlus R package was used to process the aforementioned analysis, and 1000 repeats were performed to ensure the stability of the classification (Wilkerson and Hayes, 2010). In order to determine the differences in biological processes between pyroptosis modification models, we performed gene set variation analysis (GSVA) enrichment analysis based on the “GSVA” R package. The gene set of “c2.cp.kegg. V6.4. symbols” was obtained from the MSIGDB online tool for GSVA analysis. GSVA is a nonparametric, unsupervised method for estimating genome-enrichment variation from samples of expressed datasets.

### Generation of the PRG Signature

To quantify the pyroptosis modification pattern in single cancer, a scoring system was constructed to evaluate the pyroptosis gene marker in a single HNSCC patient, which we named PyroptosisScore. The steps to construct the signature of the pyroptosis gene signature are as follows : first, the differentially expressed genes (DEGs) determined from individual PyroptosisCluster were standardized in all HNSCC samples, and the overlapping genes were collected. By using unsupervised cluster analysis to overlap the DEGs, the patients were divided into several categories for subsequent analysis. The number and stability of gene clusters are determined by the consensus clustering algorithm. Second, univariate Cox regression analysis determined the prognostic gene in the signature. Finally, principal component analysis (PCA) was conducted to establish the PRG signature and choose the main components 1 and 2 to serve as signature scores. Thus, the PyroptosisScore was acquired based on the formula (Zeng et al., 2019): *PyroptosisScore* = ∑(PC1i+PC2i) . Here, i means the expression value of phenotype PRGs.

### Estimation of TME Cell Infiltration

In order to quantify the subsets of tumor-infiltrating immune cells among the two groups and to assess their immune function, a single-sample gene-set enrichment analysis (ssGSEA) was applied. Enrichment scores obtained by ssGSEA analysis mirror the relative abundance of individual TME-infiltrated cells in every subject. Spearman correlation analysis was applied to analyze the connection between the risk score values and the TIICs (CIBERSORT, TIMER, XCELL, QUANTISEQ, EPIC, and MCPcounter algorithms). The correlation coefficients of the outcomes were demonstrated in a lollipop diagram by using R ggplot2 packages.

### Construction of the Pyroptosis-Related lncRNA Signature Based on TCGA

The prognostic value of pyroptosis-related lncRNAs determined by univariate and multivariate Cox regression analyses and the significant lncRNA were selected as characteristic lncRNAs. The prognostic signature as risk score = e^sum (lncRNA’s expression×coefficient)^. The risk score of individual patients was also counted. Based on the median risk score value, the set was divided into two (high-risk and low-risk) groups, and ROC curves were used to predict the accuracy of prognostic signatures. The pyroptosis-related lncRNA signature on patient survival was analyzed by applying the Kaplan–Meier curve.

## Statistical Analysis

The normally distributed variables and the non-normally distributed variables were performed by the unpaired student’s t-test and the Wilcoxon test, respectively. The signature and clinicopathological connections were assessed based on the chi-squared test. Logistic regression analyses to determine if the signature can be used as an independent clinical prognostic predictor. The “surv-cutpoint” function repeatedly tested all possible cut points to find the maximum rank statistic, which was classified as PyroptosisScore and then divided the patients into the high-PyroptosisScore group and the low-PyroptosisScore group based on the maximum selected log-rank statistic to reduce the batch effect of the calculation. The CNV landscape of 33 PRGs in 23 pairs of chromosomes used the R package of RCircos. The analysis of mutations in patients with high-and low-PyroptosisScore subtypes were based on the waterfall function of the maftools R package in the TCGA-HNSCC cohort. For each analysis, the statistically significant setting was *p* < 0.05.

## Results

### Landscape of the Genetic Variation of 33 PRGs

**FIGURE 1:**
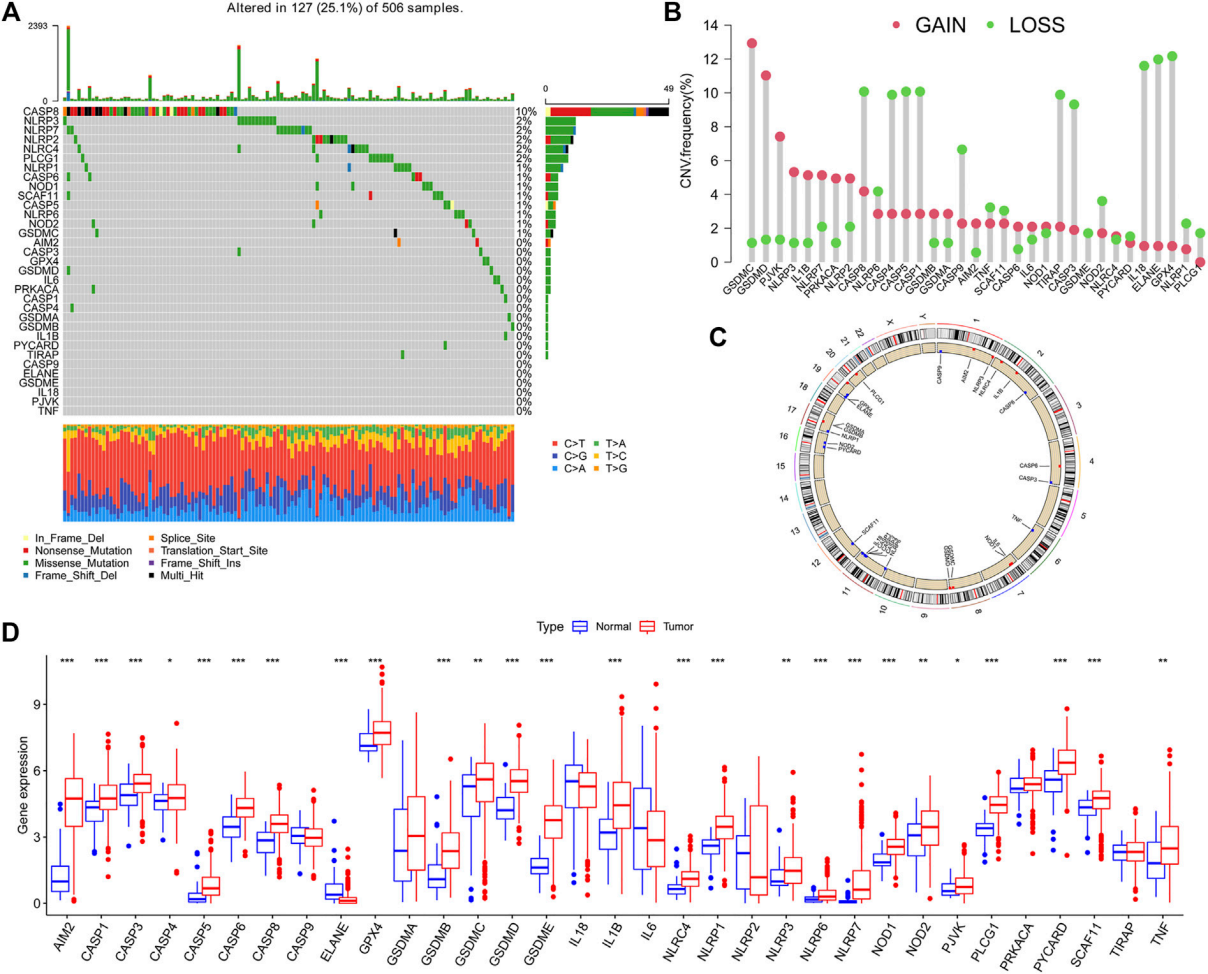
Landscape of genetic and expression variations of pyroptosis-related genes (PRGs) in HNSCC patients. **(A)**. Summarized view of the occurrence of CNV and somatic mutations of 33 PRGs in TCGA-HNSCC. **(B)**. Analysis of theCNV alteration frequency displayed that CNV changes were common in 33 PRGs, and the frequency of the copy number gain and loss was similar. **(C)**. Location of the CNV alteration of these genes on chromosomes. **(D)**. Expression of PRGs between normal and tumor in TCGA-HNSCC.

The summarized occurrence of CNV and somatic mutations of 33 PRGs in TCGA-HNSCC is shown in Figure 1A. Among 506 samples, 127 experienced mutations with a frequency of 25.1% and CASP8 demonstrated the highest mutation frequency, and most samples experienced nonsense mutation. The analysis of the CNV alteration frequency displayed that CNV changes were common in 33 PRGs, and the frequency of copy number gain and loss was similar, while GSDMC and GSDMD had a general frequency of CNV gain, and IL18, ELANE, and GPX4 had a widespread frequency of CNV loss (Figure 1B). Meanwhile, the location of the CNV alteration of these genes on chromosomes is shown in Figure 1C. The expression of these genes between normal and tumor cells in TCGA-HNSCC is shown in Figure 1D. In general, PRGs with amplified CNV were significantly higher expressed in tumor tissues and vice versa. The aforementioned analysis revealed a high level of heterogeneity in the genetic and expression patterns of the pyroptosis in HNSCC, indicating that pyroptosis plays a key role in the tumor occurrence and development.

### Pyroptosis Patterns in HNSCC

**FIGURE 2:**
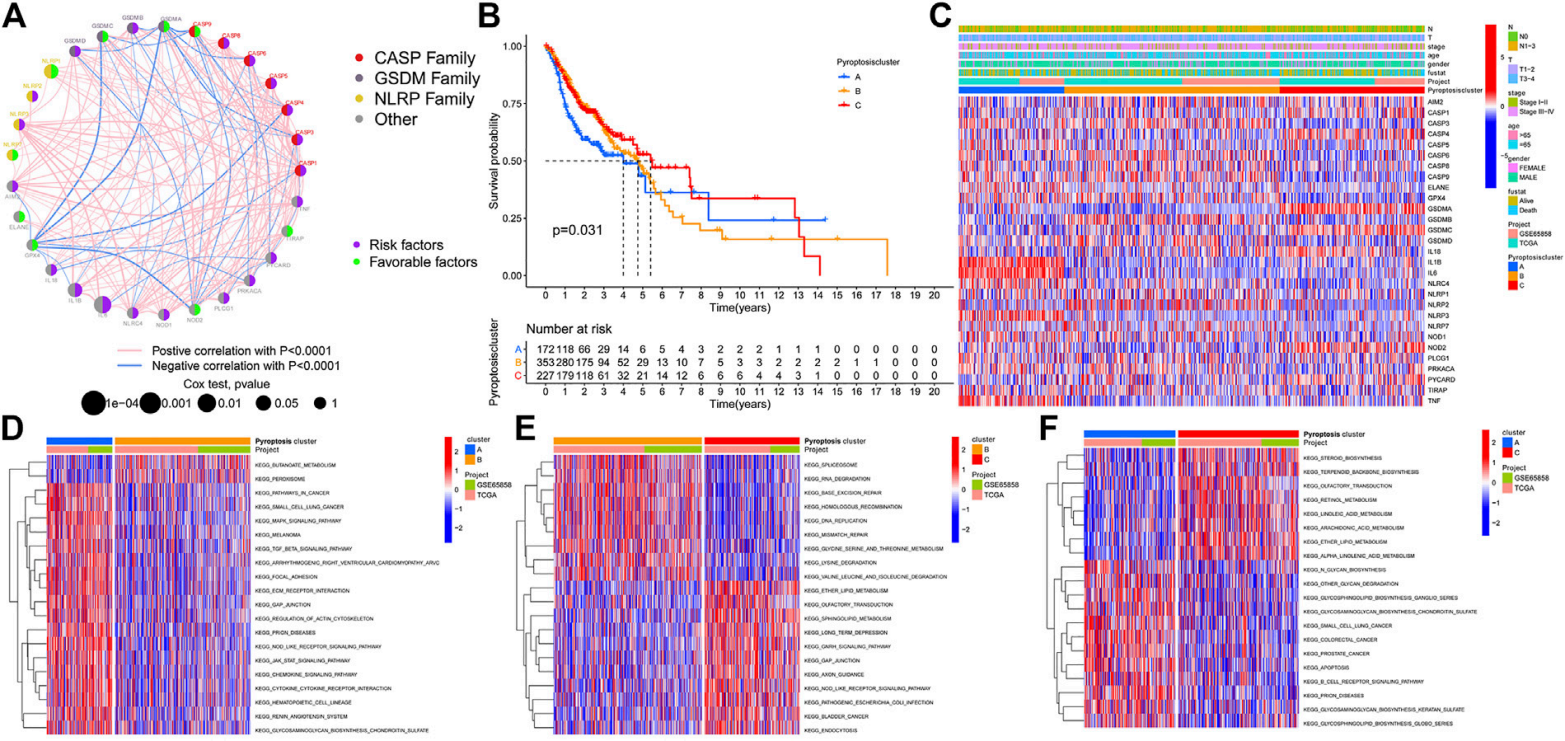
Pyroptosis patterns and biological function in HNSCC. **(A)**. Comprehensive landscape of 33 PRG gene interactions, regulator relation, and their prognosis for HNSCC patients. **(B)**. Cluster-B model indicated a particularly outstanding survival benefit. **(C)**. Heatmap for the association between PRGs and clinicopathological manifestations. **(D)**. PyroptosisCluster-A biological function. **(E)**. PyroptosisCluster-B biological function. **(F)**. PyroptosisCluster-C biological function.

Tumor cells avoid host immunosurveillance by upregulating tumor mutational burden (TMB), while TMB in turn as an indicator of immunological reaction and tumor behavior. We then merged the transcriptome data of TCGA-HNSCC and GSE65858 and obtained the mRNA expression data of 33 PRGs. A univariate Cox regression analysis determined the prognostic values of these genes in HNSCC (Supplementary Figure S1). We noticed that the expression of most genes in the CASP family affects the prognosis of patients. Thus, as CASP8 demonstrated the highest mutation frequency, we next explored the correlation between the expression of CASP8 and gene mutant and wild types. Compared with wild-type tumors, CASP1 was significantly upregulated, and IL18 was significantly downregulated in CASP8 mutant tumors (Supplementary Figure S2). Meanwhile, we also demonstrated the expressions of four (CASP1, CASP4, CASP8, and CASP9) CASP family genes based on the Human Protein Atlas database (Supplementary Figure S3). The comprehensive landscape of 33 PRG gene interactions, regulator relation, and their prognosis for HNSCC patients were portrayed within the regulator network Figure 2A. The aforementioned results suggested that pyroptosis may act as an important role in TME cell infiltrating characterization among single tumors. Based on the expressions of 33 PRGs, the ConsensusClusterPlus R package was applied to classify patients. Finally, three different clusters were identified by the unsupervised clustering method, involving 172 cases of model A, 354 cases of model B, and 227 cases of model C. The cluster B model indicated a particularly outstanding survival benefit (Figure 2B). In addition, the heatmap for the association between PRGs and clinicopathological manifestations is also analyzed Figure 2C.

### Cluster and Biological Function

**FIGURE 3:**
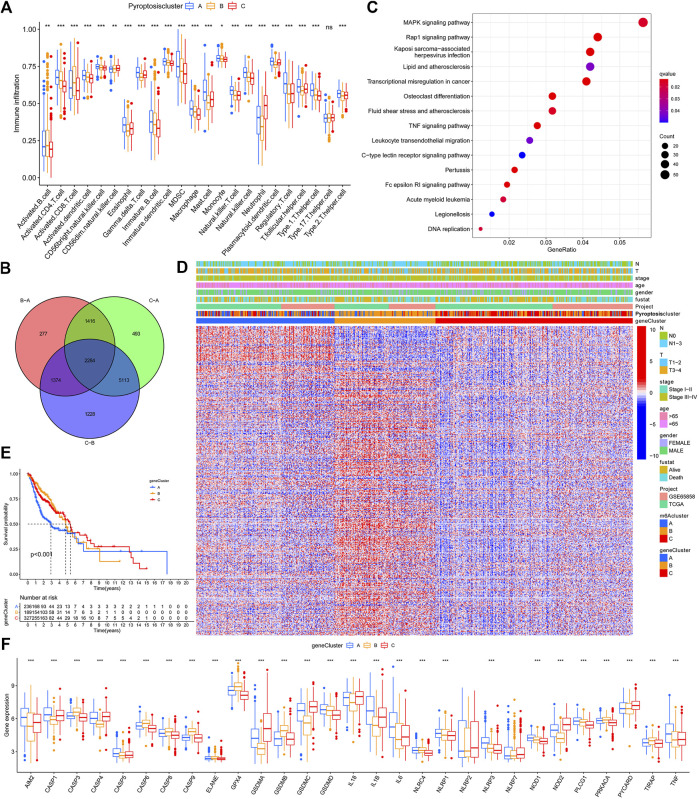
TME cell infiltration characteristics and functional annotation. **(A)**. Contrast element differences of immune cells between the clusters A, B, and C based on the ssGSEA. **(B)**. Venn diagram to identify the co-expressed genes. **(C)**. KEGG results based on the identified 2284 genes. **(D)**. Heatmap for the association between GeneClusters and clinicopathological manifestations. **(E)**. Gene cluster C demonstrated the outcome of poorer prognosis. **(F)**. Expression of PRGs between clusters A, B, and **(C)**.

GSVA enrichment analysis investigates the biological function of these different clusters. PyroptosisCluster-A was mainly enriched in pathways related to immune activation such as the cytokine–cytokine receptor interaction, chemokine signaling pathway, Toll-like receptor signaling pathways, and cell adhesion molecules cams (Figure 2D and Supplementary Table S2). PyroptosisCluster-B mostly included enrichment pathways linked to cancer degradation repairs such as DNA/RNA degradation, base excision repair, and mismatch repair (Figure 2E and Supplementary Table S3). However, PyroptosisCluster-C was prominently correlated with metabolism pathways such as linoleic acid metabolism, arachidonic acid metabolism, and ether lipid metabolism (Figure 2F and Supplementary Table S4). We then applied the ssGSEA approach to contrast the element differences of immune cells between the three clusters Figure 3A. The result suggested that there were significant differences in the compositions of immune cell types among the three clusters, which indicated that pyroptosis influences infiltrating cell types of tumors through a different biological process. We next identified the DEGs in each cluster and used the Venn diagram to identify the co-expressed genes. Finally, 2284 co-expressed genes in three clusters were identified using the limma package (Figure 3B). KEGG analysis showed that these 2284 genes mainly enriched in pathways related to carcinogenic activation pathways such as the TNF signaling pathway, Rap1 signaling pathway, Fc epsilon RI signaling pathway, transcriptional misregulation in cancer, and MAPK signaling pathway (Figure 3C and Supplementary Table S5). These genes displayed enrichment of biological process significance associated with carcinogenic pathways, which committed once more that pyroptosis played a key role in tumor occurrence.

### Construction of the Pyroptosis Gene Signatures and Functional Annotation

**FIGURE 4:**
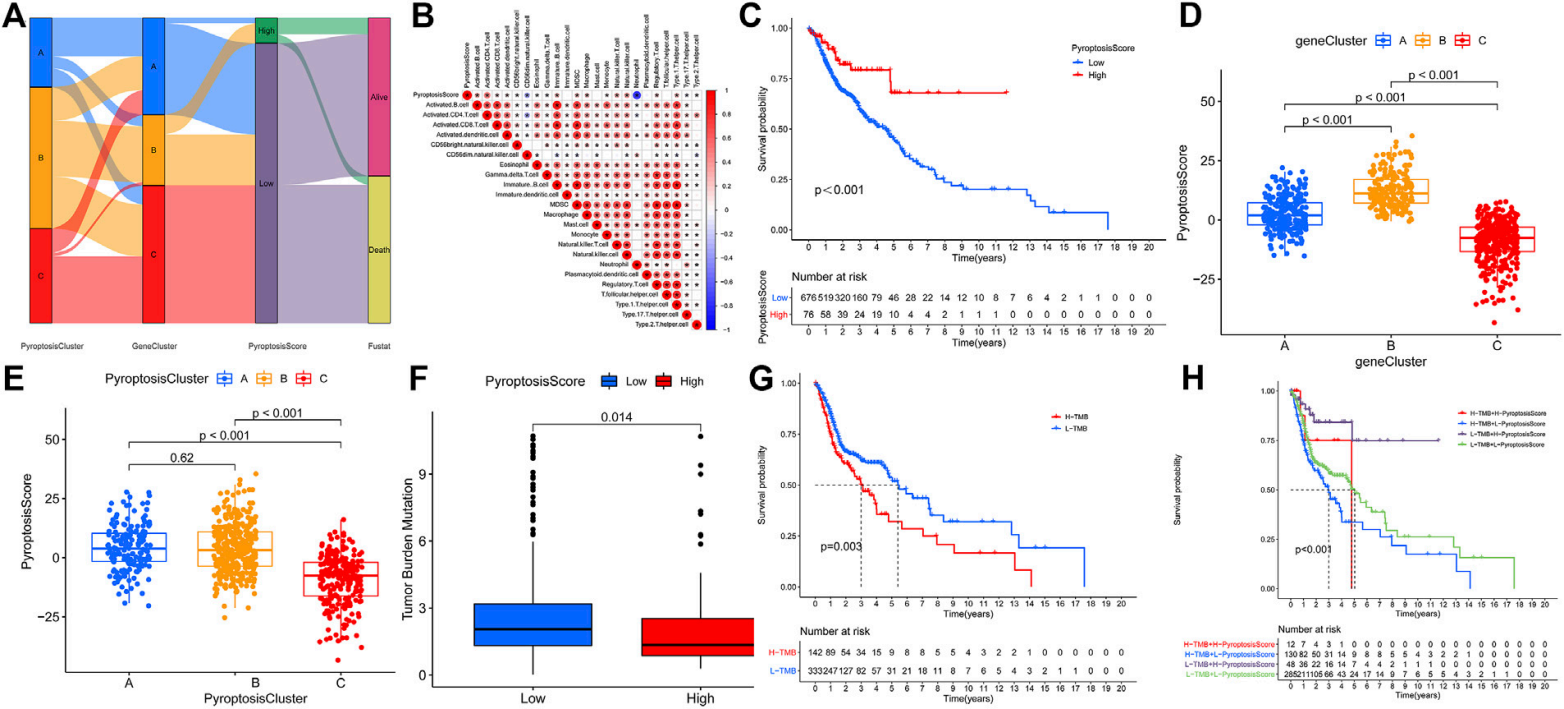
Construction of the pyroptosis gene signatures. **(A)**. ggalluvial diagram was used to visualize the relationship between PyroptosisScore, PyroptosisCluster, and GeneCluster. **(B)**. Correlation between the infiltrating cell types and the PyroptosisScore. **(C)**. Value of the PyroptosisScore in predicting patients’ prognosis. **(D)**. Connection between the PyroptosisScore and pyroptosis GeneClusters. **(E)**. Connection between the PyroptosisScore and PyroptosisCluster. **(F)**. Connection between the high/low score group and TMB. **(G)**. High TMB group suggested a poorer prognosis. **(H)**. Prognosis results in different TMB when combined with the PyroptosisScore.

To further explore the possible regulatory mechanism, unsupervised cluster analysis was conducted based on the acquired 2284 pyroptosis phenotypic-related genes to classify patients into distinct genomic subtypes. Three distinct pyroptosis genomic phenotypes were determined and named as pyroptosis gene clusters A/B/C. The analysis showed that three different gene clusters had different characteristic genes (Figure 3D). Gene cluster C demonstrated the outcome of a poorer prognosis Figure 3E. In the three pyroptosis gene clusters, the main differences in the expression of PRGs were noticed, which was inconsistent with the prospective outcomes of PyroptosisCluster patterns Figure 3F. Considering the individual heterogeneity, a scoring system to quantify the pyroptosis pattern in each patient was constructed based on these phenotype-related genes, which we named as PyroptosisScore. The ggalluvial diagram was used to visualize the relationship between PyroptosisScore, PyroptosisCluster, and GeneCluster Figure 4A. The correlation between the infiltrating cell types and the PyroptosisScore is presented in Figure 4B. Then, the patients were divided into low- or high-PyroptosisScore groups based on the median value. The value of PyroptosisScore in predicting patients’ prognosis was also explored, and patients with high PyroptosisScore indicated a bad survival outcome Figure 4C. The Kruskal–Wallis test determined a significant difference in PyroptosisScore among pyroptosis GeneClusters and PyroptosisCluster (Figure 4D,E). PyroptosisCluster A and B significantly increased when compared to PyroptosisCluster A which indicated that cancer-related pathways indicated high scores Figure 2D–F. Studies reported that the tumor mutational burden (TMB) plays an important role in tumor prognosis. Thus, we next explored the connection between the high/low score group and TMB, and the results have shown that the low score group demonstrated high TMB (Figure 4F). Interestingly, while the high TMB group suggested a poorer prognosis, when high TMB was combined with a low PyroptosisScore, there was a beneficial outcome indicated which may result in heterogeneity and needs more in-depth research studies (Figure 4G,H).

### Characteristics of the PyroptosisScore

**FIGURE 5:**
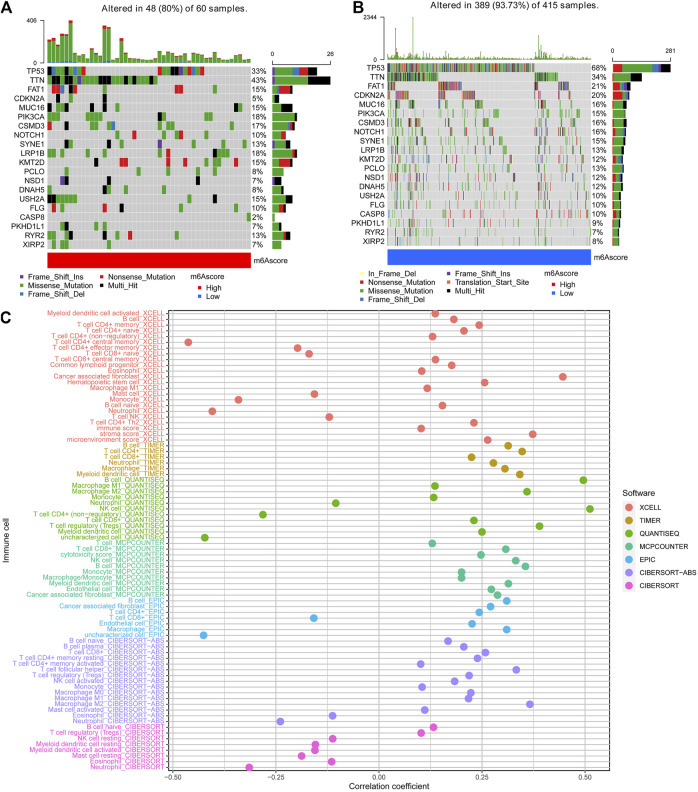
Characteristics of the PyroptosisScore signatures. **(A)**. Distribution of somatic mutations in the high-PyroptosisScore group. **(B)**. Distribution of somatic mutations in the low-PyroptosisScore group. **(C)**. Detailed Spearman correlation analysis performed using different algorithms.

**FIGURE 6:**
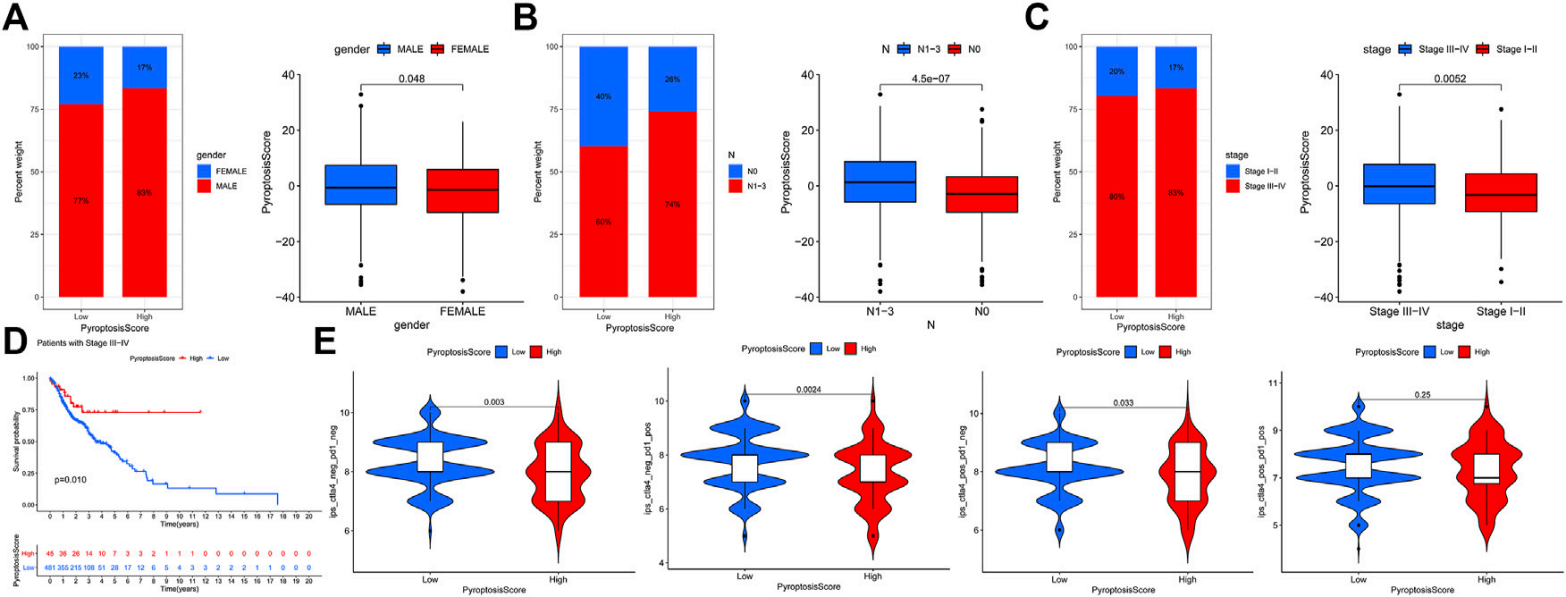
Relationship between the clinical characteristics and PyroptosisScore. **(A)**. Gender, **(B)**. N stage, and **(C)**. tumor stage. **(D)**. High score group means a bad prognosis in stage III-IV patients. **(E)**. Immunophenoscore (IPS) function is based on The *Cancer* Immunome Atlas database to predict the responsiveness to *CTLA-4* and *PD-1*.

We then used the maftools package to analyze the differences in the distribution of somatic mutations among low and high PyroptosisScores in the TCGA-HNSCC cohort. The results indicated that the low-PyroptosisScore group experienced increased extensive TMB than the high group, with the most significant mutated genes being TP53 and TTN Figure 5A,B. A detailed Spearman correlation analysis was also performed using different algorithms, a lollipop shape to exhibit the result, as shown in Figure 5C. From the results, we can find that most immune cells have a positive correlation with the PyroptosisScore. The relationship between the clinical characteristics and PyroptosisScore was also explored, and we found that a high score may be related to an advanced tumor stage Figure 6A–C. In addition, a high score group means a bad prognosis in stage III-IV patients Figure 6D. We finally used the immunophenoscore (IPS) function based on The Cancer Immunome Atlas database to predict the responsiveness to CTLA-4 and PD-1. The results showed that the high- and low-score groups had a good response to PD-1 and CTL single positive or double negative (Figure 6E), which can provide potentially useful insights for further analysis. Meanwhile, we also found a statistically significant difference in OS between immunotherapy and risk scores based on the IMvigor210 cohort (Mariathasan Turley Nickles, 2018) (Supplementary Figure S4A). In addition, the AUC value of the PyroptosisScore signature was 0.735, indicating that the prediction accuracy is moderate (Supplementary Figure S4B).

### Pyroptosis-Related lncRNA

Finally, we tried to find some useful pyroptosis-related lncRNAs. There were 525 pyroptosis-related lncRNAs determined according to the screening criteria. The lncRNA OSER1-DT had the largest correlation coefficient, and the target gene was *GPX4* (cor = 0.65; *p* = 1.07E-59; Supplementary Table S6). Differentially expressed lncRNAs were conducted in univariate Cox analysis to screen the prognostic lncRNAs. Multivariate Cox analysis comprised 57 lncRNAs of great significance in univariate Cox analysis (Supplementary Table S7). Finally, 14 differentially expressed lncRNAs (LINC01963, AL132989.1, AC060780.1, AL451085.2, AC098487.1, PTOV1-AS2, LINC00460, AC012467.2, LINC00958, CTBP1-DT, AC116914.2, AC103702.2, AL022328.2, and AL450992.2) were selected as independent prognosis factors of HNSCC patients Supplementary Table S8. Therefore, risk scores were calculated, and prognostic signatures for lncRNAs were constructed. The expressions of these 14 lncRNAs on patient survival were analyzed next using the Kaplan–Meier curve. The results demonstrated that all these 14 lncRNA expression significance effect patient’s OS (Supplementary Figure S5). Patients with high risk have poorer survival and as the risk score increases, the number of disease patients also elevated (Supplementary Figure S6A, S5B). There was an AUC of 0.766 in the set and the 14 lncRNA-based signatures (HR:1.69; 95%CI: 1.38–2.07) and N stage (HR:1.70; 95%CI: 1.15–2.52) were established as independent prognosis factors of OS (Supplementary Figure S6C-E). In total, the signature may act on the pyroptosis-related lncRNA status of HNSCC patients and present possible biomarkers for clinical therapeutic intervention.

## Discussion

Currently, the combined application of multiple immunotherapies and the combined application of immunotherapy, radiotherapy, chemotherapy, and targeted therapy may improve the prognosis of cancer patients by inhibiting tumor progression through the synergistic action of multiple mechanisms. In cancer, pyroptosis is considered to be an autonomous tumor suppressor mechanism, which has a profound impact on the inhibition of tumor progression. Thus, inducing pyroptosis cell death or activating the related signaling pathways is suggested to be a novel way of cancer therapy. This study determined a novel and effective pyroptosis-related prognostic characterization and the TME cell infiltration dysfunction in the HNSCC patients. The analysis may help us understand the pyroptosis status and TME antitumor immune response and thus provide potential biomarkers for clinical therapeutic intervention.

As a newly discovered method of cell death, pyroptosis has two-sided effects on tumors. On the one hand, the key inflammasomes in the process of pyroptosis can promote tumor cell death and inhibit tumor cell proliferation and metastasis; on the other hand, the accumulation of inflammasomes is conducive to the formation of a microenvironment suitable for tumor cell growth, proliferation, and metastasis. Translocation of GSDMD protein is a necessary procedure for the formation of membrane pores within pyroptosis. A recent study reported that DRD2 can regulate the tumor microenvironment and facilitate M1 polarization of macrophages and activated GSDME-executed pyroptosis in breast cancer (Tan et al., 2021a). Silencing GSDME alleviates cisplatin- or doxorubicin-induced HK-2 cell pyroptosis by raising cell viability and reducing LDH release (Shen et al., 2021). Our study indicated that GSDMC and GSDMD had a general frequency of CNV amplification, and CASP8 demonstrated the highest mutation frequency. CASP8 suggested the molecular switch that controls apoptosis, pyroptosis, and necroptosis, and protects against tissue damage (Fritsch et al., 2019). Caspase‐8 can directly cleave caspase‐3 to activate apoptosis or GSDMD to trigger pyroptosis, as well as also cleaving RIPK1 and RIPK3 to inhibit necroptosis (Orning and Lien, 2021). Moreover, membrane cholesterol is essential for the activation and recruitment of caspase-8 and its non-apoptotic functions in cancer cells (Kumar et al., 2018). Until now, there is no related study about the role of CASP8 in HNSCC, and our analysis can provide useful information for the in-depth exploration.

Pyroptosis plays an antitumor role by activating the immune response. Due to the defect of the cell membrane, the pyroptotic cells liberate a large amount of cellular substance to cause a strong inflammatory response and a large infiltration of lymphocytes. Lymphocyte infiltration was significantly increased, which further induced caspase-3-independent and -dependent tumor cell pyroptosis, thus forming positive feedback to enhance the anti-tumor function (Wang et al., 2020). Colocalization of GSDMD with granzyme B was shown in the proximity of immune synapses, and GSDMD deficiency lessens the cytolytic capacity of CD8^+^ T cells (Xi et al., 2019). The study also presented that promoting tumor-localized necroptosis and pyroptosis may finally increase responses to immune checkpoint inhibitors (ICI) and tumor-infiltrating lymphocytes (Rosenbaum et al., 2021). Pyroptosis even appears to reprogram the tumor microenvironment to an immunostimulatory state (Tsuchiya, 2021). In our study, biological function analysis revealed that pyroptosis PyroptosisCluster was mainly enriched in pathways related to immunity and cancer, and ssGSEA also suggested that there were significant differences in the compositions of immune cell types among the clusters.

Long non-coding RNA (lncRNA) participates in numerous biological regulatory courses and thus is closely linked to the incidence, progress, and metastasis of cancers. Recent studies have indicated that lncRNAs participated in the malignant phenotypes of tumors not only by genomic or transcriptomic alterations but also through changing the immune microenvironment. A recent study suggested that silencing of lncRNA HOTTIP results in the inhibition of cell proliferation and NLRP1 inflammasome-mediated pyroptosis in ovarian cancer (Tan et al., 2021b). LncRNA ADAMTS9-AS2 served as a tumor suppressor and improved cisplatin sensitivity in gastric cancer cells through activating NLRP3-mediated pyroptotic cell death by sponging miR-223-3p (Ren et al., 2020). In our study, we identified 14 differentially expressed lncRNAs (LINC01963, AL132989.1, AC060780.1, AL451085.2, AC098487.1, PTOV1-AS2, LINC00460, AC012467.2, LINC00958, CTBP1-DT, AC116914.2, AC103702.2, AL022328.2, and AL450992.2), which were chosen as independent prognosis factors of HNSCC patients. Although pyroptosis plays a critical role in tumors, the pyroptosis-related lncRNAs that affect the responsible genes have not been determined. This study integrated some lncRNA biomarkers to assess their effect on patients’ prognosis; this can contribute to the determination of novel biomarkers and accurate medical targets in HNSCC. In addition, this study can assist with prognosis prediction, diagnosis, and management strategies of patients with HNSCC. However, further independent studies are required to prove these outcomes and the predictive pyroptosis-related lncRNA. This study also provides some novel insights into cancer immunotherapies that target the genes involved in apoptosis and further reverse the undesirable TME cell infiltration characteristics, which is the transformation of “cold tumor” to “hot tumor,” and may contribute to the development of new drug combination strategies or new immunotherapeutic agents. Finally, there were limitations to this study. First, the outcomes were not validated in clinical samples. Second, the results do not provide accurate clinical data due to the relatively small number of patients. Finally, it is unclear whether some genes (such as CASP9 and NLRP7) are pyroptosis-related genes, so we should be cautious when interpreting our results. Although this research explored the probability of establishing a prognostic prediction model, improvement is required.

## Conclusion

This work suggests the pyroptosis features and the potential mechanisms on the tumor microenvironment. The exploration may assist in identifying novel biomarkers and help patients in predicting prognosis, clinical diagnosis, and management.

## Data Availability

The datasets presented in this study can be found in online repositories. The names of the repository/repositories and accession number(s) can be found in the article/Supplementary Material.

## Abbreviations

CNV, copy number variation; GEO, Gene Expression Omnibus; GSDMD, gasdermin protein D; HNSCC, head and neck squamous cell carcinoma; PRGs, pyroptosis-related genes; TME, tumor microenvironment; TCGA, The Cancer Genome Atlas; TPM, transcripts per kilobase million.

## References

[B1] Bishop J. A. Gagan J. Paterson C. McLellan D. Sandison A. (2021). Nonkeratinizing Squamous Cell Carcinoma of the Sinonasal Tract with DEK-AFF2. Am. J. Surg. Pathol. 45 (5), 718–720. 10.1097/pas.0000000000001596 33002918

[B2] Cui J. Zhou Z. Yang H. Jiao F. Li N. Gao Y. (2019). MST1 Suppresses Pancreatic Cancer Progression via ROS-Induced Pyroptosis. Mol. Cancer Res. 17 (6), 1316–1325. 10.1158/1541-7786.mcr-18-0910 30796177

[B3] Ferlay J. Shin H.-R. Bray F. Forman D. Mathers C. Parkin D. M. (2010). Estimates of Worldwide Burden of Cancer in 2008: GLOBOCAN 2008. Int. J. Cancer 127, 2893–2917. 10.1002/ijc.25516 21351269

[B4] Fritsch M. Günther S. D. Schwarzer R. Albert M.-C. Schorn F. Werthenbach J. P. (2019). Caspase-8 Is the Molecular Switch for Apoptosis, Necroptosis and Pyroptosis. Nature 575 (7784), 683–687. 10.1038/s41586-019-1770-6 31748744

[B5] Geng X. Zhang Y. Zeng Z. Zhu Z. Wang H. Yu W. (2021). Molecular Characteristics, Prognostic Value, and Immune Characteristics of m6A Regulators Identified in Head and Neck Squamous Cell Carcinoma. Front. Oncol. 11, 629718. 10.3389/fonc.2021.629718 33816266 PMC8014089

[B6] Hartigan J. A. W. M. (1979). Algorithm AS 136: a K-Means Clustering Algorithm. Appl. Stat. 28, 9. 10.2307/2346830

[B7] Hitt M. M. Allday M. J. Hara T. Karran L. Jones M. D. Busson P. (1989). EBV Gene Expression in an NPC-Related Tumour. EMBO J. 8 (9), 2639–2651. 10.1002/j.1460-2075.1989.tb08404.x 2479554 PMC401270

[B8] Huang Z. Zhang Q. Wang Y. Chen R. Wang Y. Huang Z. (2020). Inhibition of Caspase-3-Mediated GSDME-Derived Pyroptosis Aids in Noncancerous Tissue Protection of Squamous Cell Carcinoma Patients during Cisplatin-Based Chemotherapy. Am. J. Cancer Res. 10 (12), 4287–4307. 33415000 PMC7783734

[B9] Jacobs C. Lyman G. Velez-García E. Sridhar K. S. Knight W. Hochster H. (1992). A Phase III Randomized Study Comparing Cisplatin and Fluorouracil as Single Agents and in Combination for Advanced Squamous Cell Carcinoma of the Head and Neck. Jco 10, 257–263. 10.1200/jco.1992.10.2.257 1732427

[B10] Jorgensen I. Rayamajhi M. Miao E. A. (2017). Programmed Cell Death as a Defence against Infection. Nat. Rev. Immunol. 17, 151–164. 10.1038/nri.2016.147 28138137 PMC5328506

[B11] Karki R. Kanneganti T.-D. (2019). Diverging Inflammasome Signals in Tumorigenesis and Potential Targeting. Nat. Rev. Cancer 19 (4), 197–214. 10.1038/s41568-019-0123-y 30842595 PMC6953422

[B12] Kumar M. Irungbam K. Kataria M. (2018). Depletion of Membrane Cholesterol Compromised Caspase-8 Imparts in Autophagy Induction and Inhibition of Cell Migration in Cancer Cells. Cancer Cell Int. 18, 23. 10.1186/s12935-018-0520-4 29467593 PMC5819249

[B13] Liu J. Yao L. Zhang M. Jiang J. Yang M. Wang Y. (2019). Downregulation of LncRNA-XIST Inhibited Development of Non-small Cell Lung Cancer by Activating miR-335/SOD2/ROS Signal Pathway Mediated Pyroptotic Cell Death. Aging 11, 7830–7846. 10.18632/aging.102291 31553952 PMC6781979

[B14] Man S. M. Kanneganti T.-D. (2015). Regulation of Inflammasome Activation. Immunol. Rev. 265 (1), 6–21. 10.1111/imr.12296 25879280 PMC4400844

[B15] Mariathasan S. Turley S. J. Nickles D. Castiglioni A. Yuen K. Wang Y. TGFβ Attenuates Tumour Response to PD-L1 Blockade by Contributing to Exclusion of T Cells. Nature. 2018 Feb 22;554(7693):544–548. 10.1038/nature25501 29443960 PMC6028240

[B16] Orning P. Lien E. (2021). Multiple Roles of Caspase‐8 in Cell Death, Inflammation, and Innate Immunity. J. Leukoc. Biol. 109 (1), 121–141. 10.1002/jlb.3mr0420-305r 32531842 PMC8664275

[B17] Peyrade F. Borel C. Daste A. Even C. Saada-Bouzid E. Guigay J. (2021). Systemic Treatment of Metastatic Squamous Cell Carcinoma of the Head and Neck: Proposal for Management Changes. Curr. Opin. Oncol. 33 (3), 160–167. 10.1097/cco.0000000000000738 33782359

[B18] Qiu S. Liu J. Xing F. (2017). 'Hints' in the Killer Protein Gasdermin D: Unveiling the Secrets of Gasdermins Driving Cell Death. Cell Death Differ. 24 (4), 588–596. 10.1038/cdd.2017.24 28362726 PMC5384029

[B19] Ren N. Jiang T. Wang C. Xie S. Xing Y. Piao D. (2020). LncRNA ADAMTS9-AS2 Inhibits Gastric Cancer (GC) Development and Sensitizes Chemoresistant GC Cells to Cisplatin by Regulating miR-223-3p/NLRP3 axis. Aging 12 (11), 11025–11041. 10.18632/aging.103314 32516127 PMC7346038

[B20] Rosenbaum S. R. Wilski N. A. Aplin A. E. (2021). Fueling the Fire: Inflammatory Forms of Cell Death and Implications for Cancer Immunotherapy. Cancer Discov. 11 (2), 266–281. 10.1158/2159-8290.cd-20-0805 33451983 PMC7858229

[B21] Rühl S. Shkarina K. Demarco B. Heilig R. Santos J. C. Broz P. (2018). ESCRT-Dependent Membrane Repair Negatively Regulates Pyroptosis Downstream of GSDMD Activation. Science 362 (6417), 956–960. 30467171 10.1126/science.aar7607

[B22] Shen X. Wang H. Weng C. Jiang H. Chen J. (2021). Caspase 3/GSDME-dependent Pyroptosis Contributes to Chemotherapy Drug-Induced Nephrotoxicity. Cell Death Dis. 12 (2), 186. 10.1038/s41419-021-03458-5 33589596 PMC7884686

[B23] Tan C. Liu W. Zheng Z. H. Wan X. G. Lncrna Hottip Inhibits Cell Pyroptosis by Targeting miR-148a-3p/AKT2 axis in Ovarian Cancer. Cell Biol. Int. 2021 Mar 12. 10.1002/cbin.11588 33710684

[B24] Tan Y. Sun R. Liu L. Yang D. Xiang Q. Li L. (2021). Tumor Suppressor DRD2 Facilitates M1 Macrophages and Restricts NF-Κb Signaling to Trigger Pyroptosis in Breast Cancer. Theranostics 11 (11), 5214–5231. 10.7150/thno.58322 33859743 PMC8039962

[B25] Tsuchiya K. (2021). Switching from Apoptosis to Pyroptosis: Gasdermin-Elicited Inflammation and Antitumor Immunity. Ijms 22 (1), 426. 10.3390/ijms22010426 PMC7794676 33406603

[B26] Wang B. Yin Q. (2017). AIM2 Inflammasome Activation and Regulation: A Structural Perspective. J. Struct. Biol. 200 (3), 279–282. 10.1016/j.jsb.2017.08.001 28813641 PMC5733693

[B27] Wang Q. Wang Y. Ding J. Wang C. Zhou X. Gao W. (2020). A Bioorthogonal System Reveals Antitumour Immune Function of Pyroptosis. Nature 579 (7799), 421–426. 10.1038/s41586-020-2079-1 32188939

[B28] Wilkerson M. D. Hayes D. N. (2010). ConsensusClusterPlus: a Class Discovery Tool with Confidence Assessments and Item Tracking. Bioinformatics 26, 1572–1573. 10.1093/bioinformatics/btq170 20427518 PMC2881355

[B29] Xi G. Gao J. Wan B. Zhan P. Xu W. Lv T. (2019). GSDMD Is Required for Effector CD8+ T Cell Responses to Lung Cancer Cells. Int. Immunopharmacol. 74, 105713. 10.1016/j.intimp.2019.105713 31276977

[B30] Xia X. Wang X. Cheng Z. Qin W. Lei L. Jiang J. (2019). The Role of Pyroptosis in Cancer: Pro-cancer or Pro-"host"? Cell Death Dis. 10 (9), 650. 10.1038/s41419-019-1883-8 31501419 PMC6733901

[B31] Zeng D. Li M. Zhou R. Zhang J. Sun H. Shi M. (2019). Tumor Microenvironment Characterization in Gastric Cancer Identifies Prognostic and Immunotherapeutically Relevant Gene Signatures. Cancer Immunol. Res. 7, 737–750. 10.1158/2326-6066.cir-18-0436 30842092

[B32] Zhang M. J. Gao W. Liu S. Siu S. P. Yin M. Ng J. C. (2020). CD38 Triggers Inflammasome-Mediated Pyroptotic Cell Death in Head and Neck Squamous Cell Carcinoma. Am. J. Cancer Res. 10 (9), 2895–2908. 33042624 PMC7539785

[B33] Zhou Z. He H. Wang K. Shi X. Wang Y. Su Y. (2020). Granzyme A from Cytotoxic Lymphocytes Cleaves GSDMB to Trigger Pyroptosis in Target Cells. Science 368, eaaz7548. 10.1126/science.aaz7548 32299851

