# Supplementary figures and images for: Pyroptosis-Related Signature and Tumor Microenvironment Infiltration Characterization in Head and Neck Squamous Cell Carcinoma
Source: Front Cell Dev Biol. 2022 May 31;10:702224. doi: 10.3389/fcell.2022.702224 (PMC9194563; doi:10.3389/fcell.2022.702224)

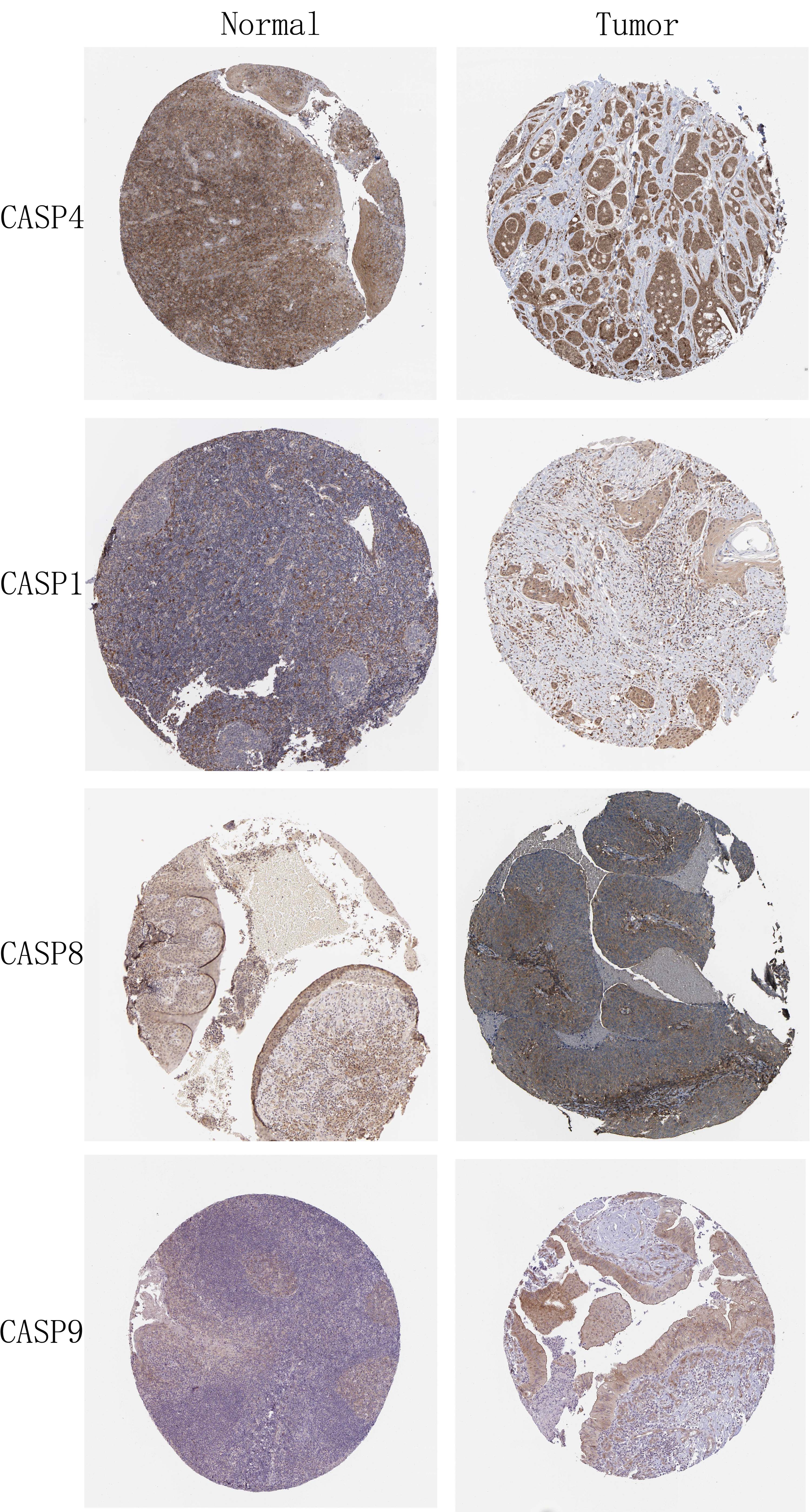

Supplement: Supplementary file 1 [file Image3.JPEG]

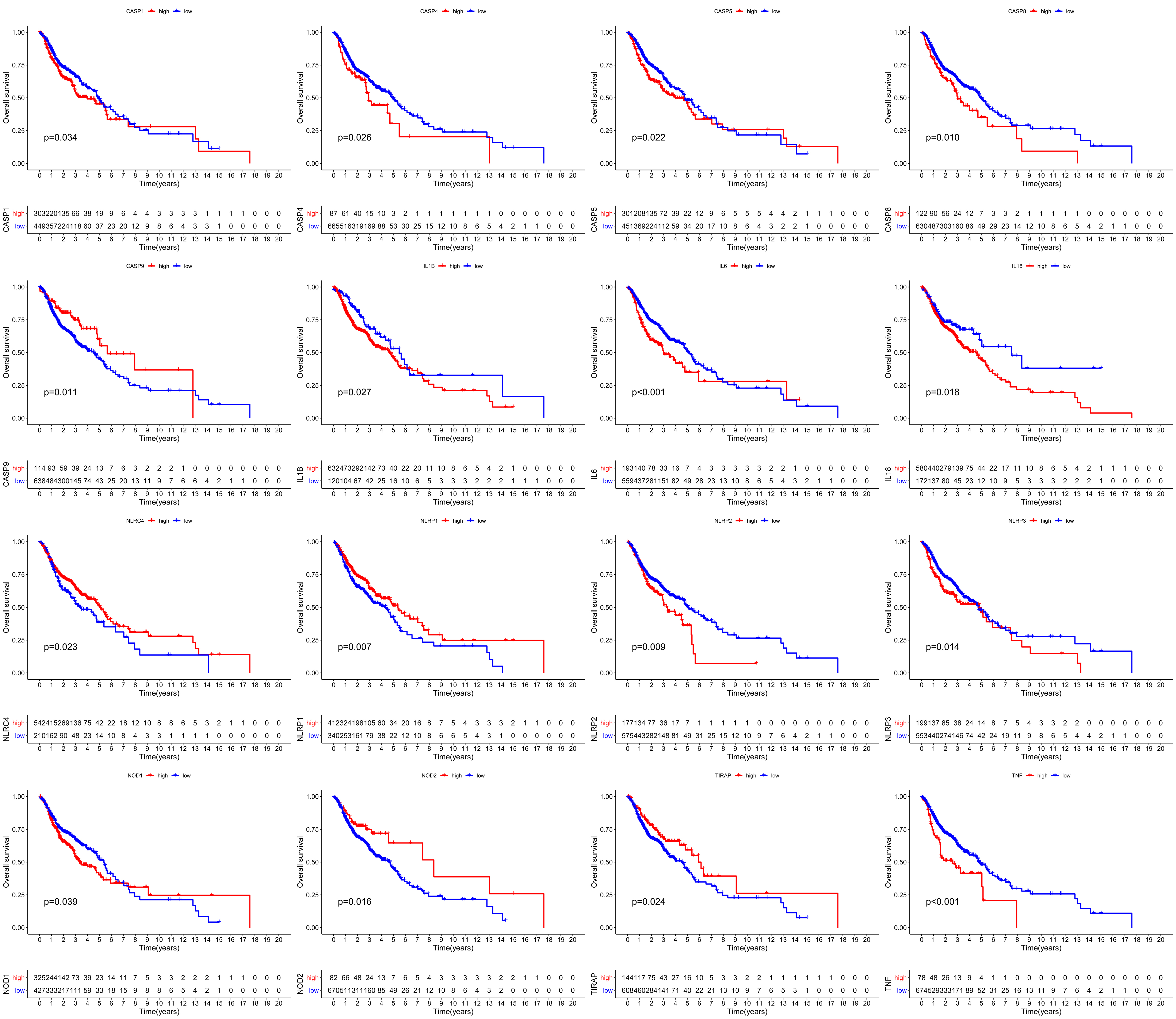

Supplement: Supplementary file 2 [file Image1.JPEG]

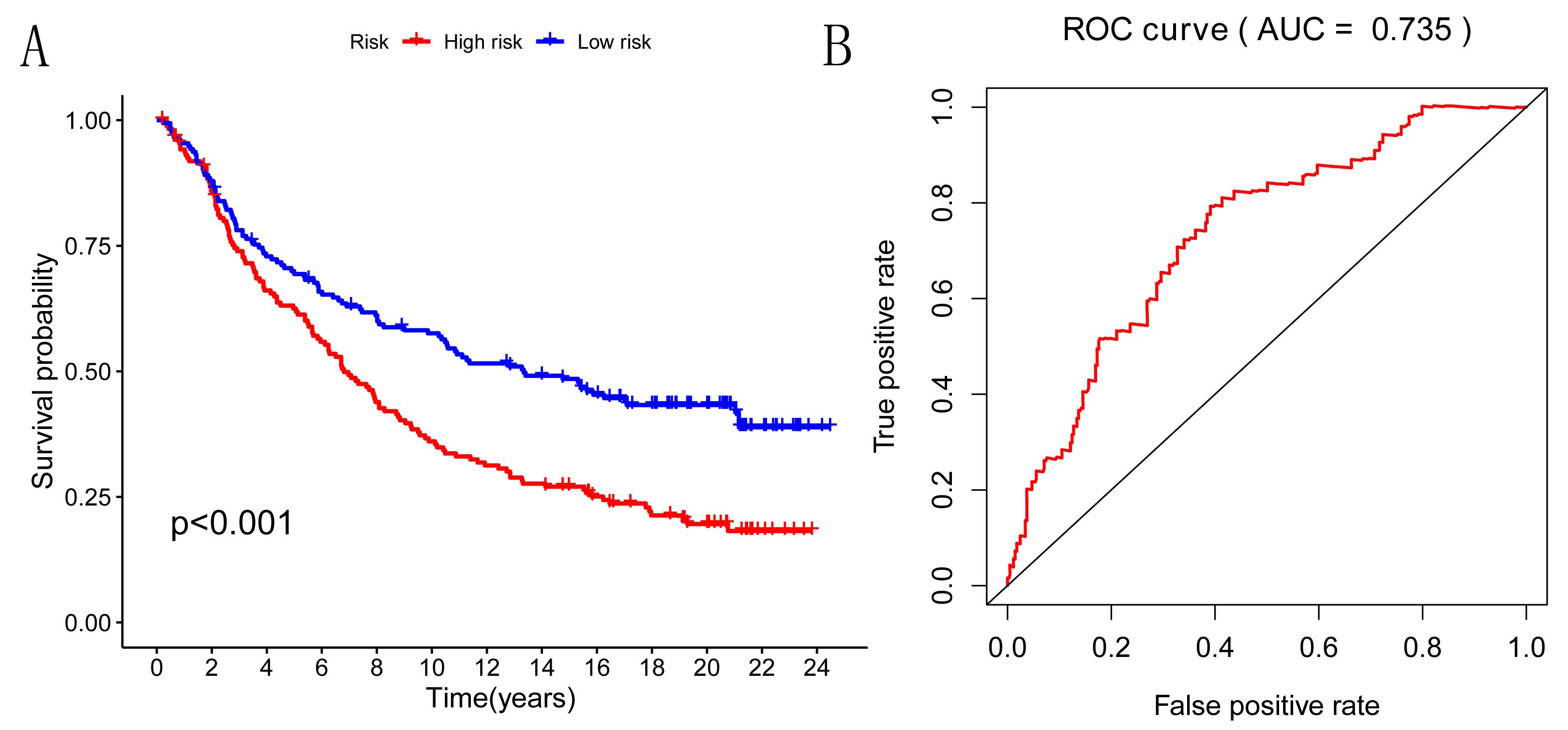

Supplement: Supplementary file 3 [file Image4.JPEG]

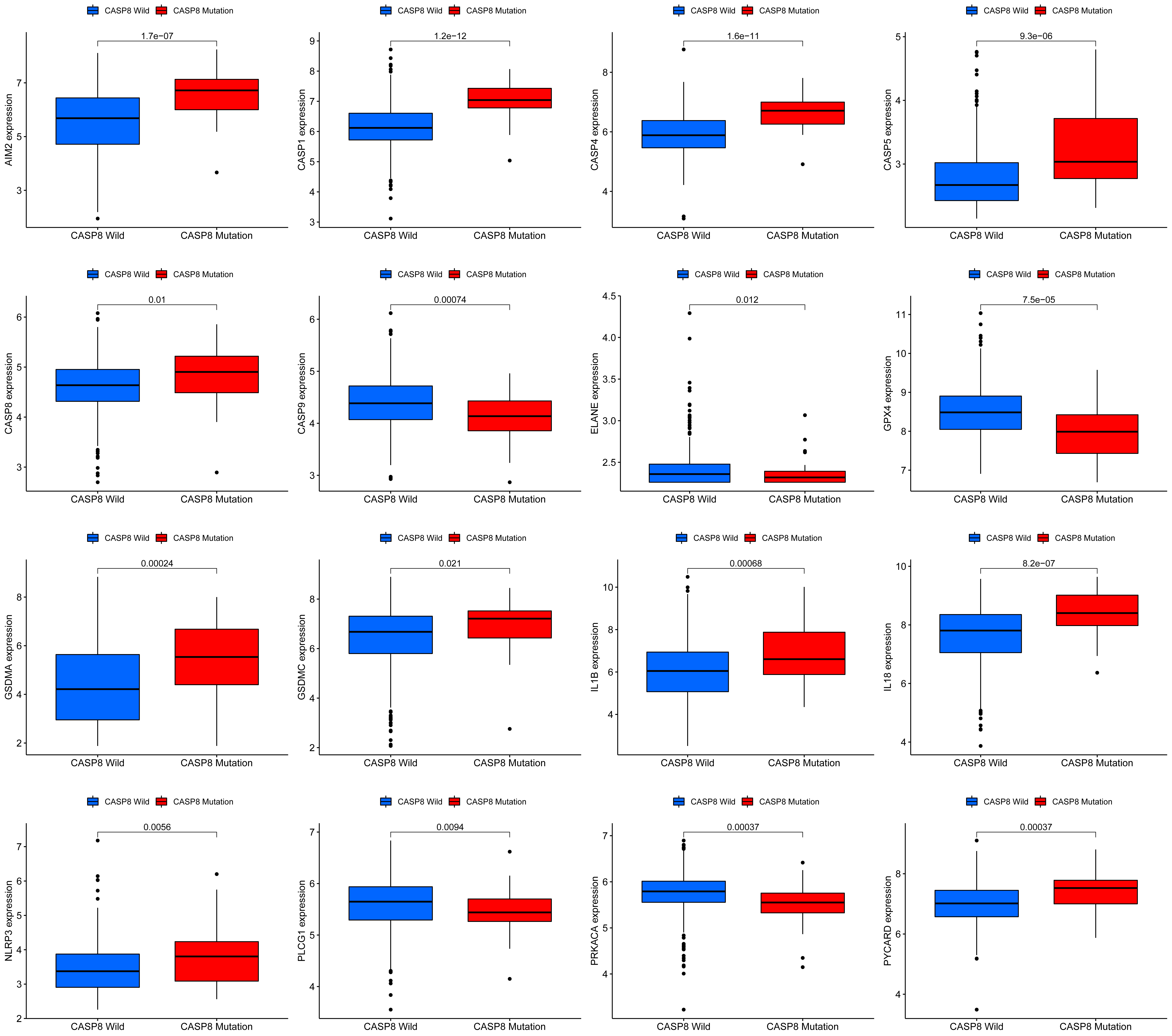

Supplement: Supplementary file 4 [file Image2.JPEG]

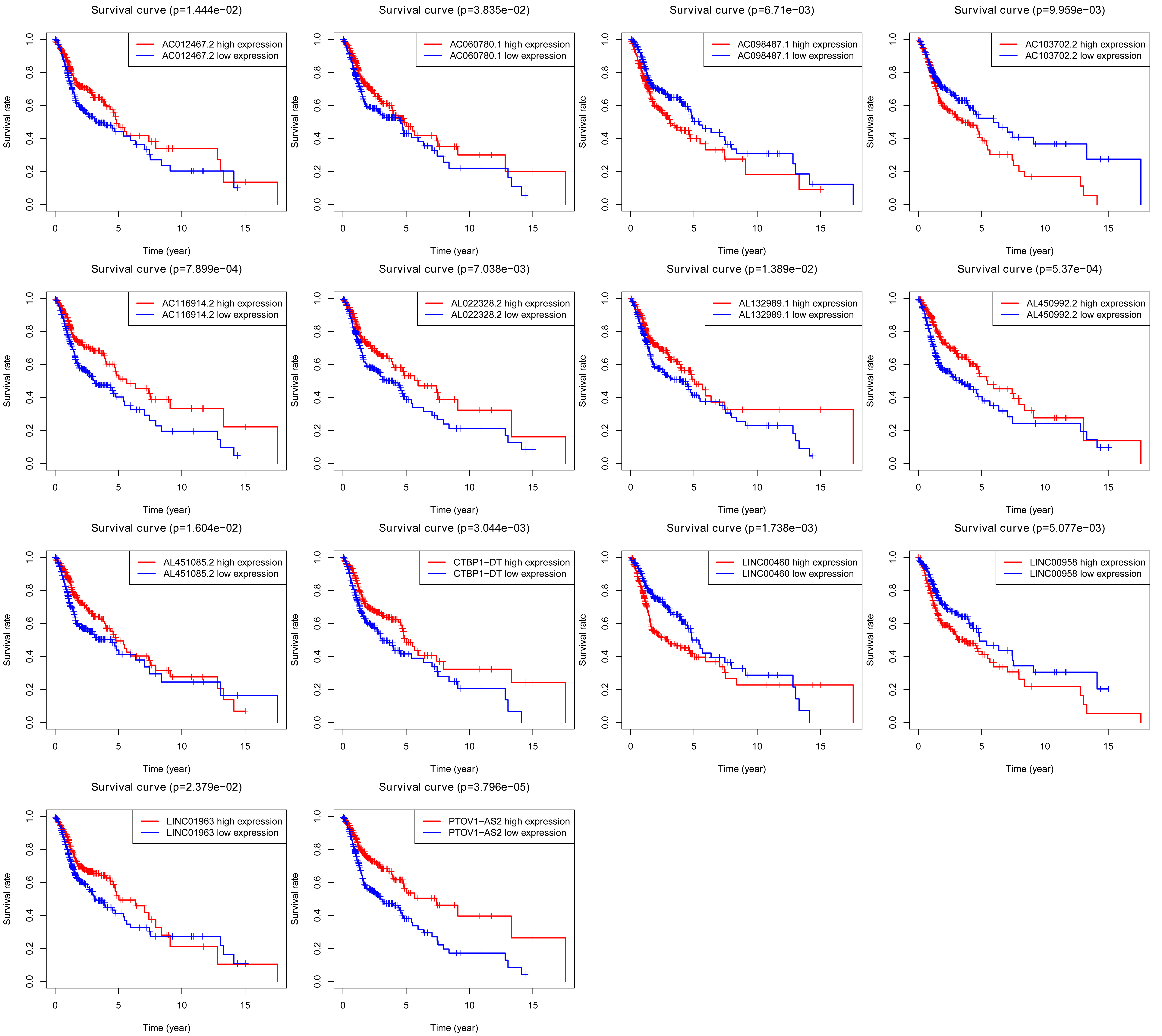

Supplement: Supplementary file 5 [file Image5.JPEG]

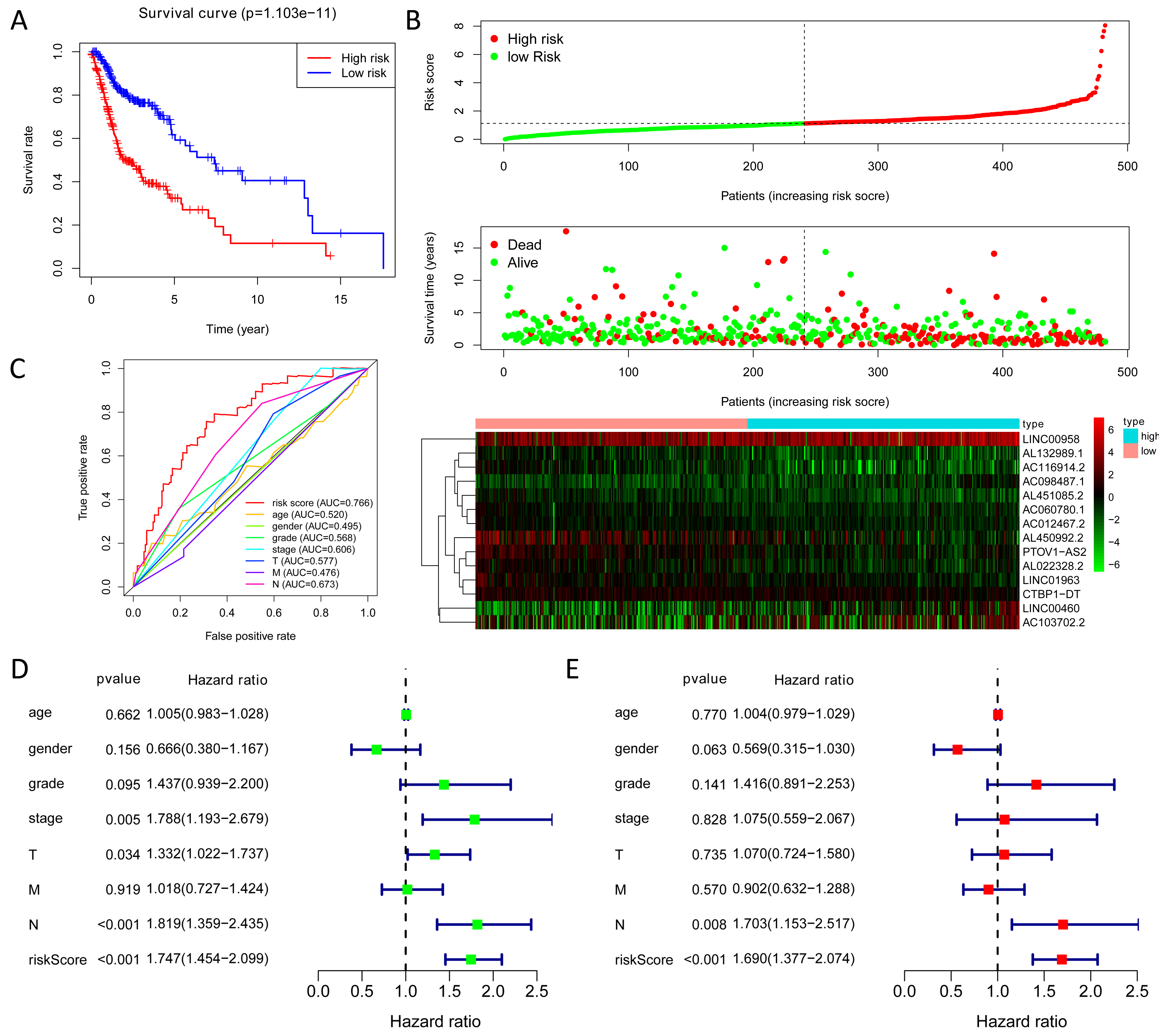

Supplement: Supplementary file 7 [file Image6.JPEG]
